# The Discrepancy between Preoperative Tumor Markers and Imaging Outcomes in Predicting Ovarian Malignancy

**DOI:** 10.3390/cancers14235821

**Published:** 2022-11-25

**Authors:** Kyung-Hwa Shin, Hyung-Hoi Kim, Hyung Joon Yoon, Eun Taeg Kim, Dong Soo Suh, Ki Hyung Kim

**Affiliations:** 1 Department of Laboratory Medicine, Pusan National University School of Medicine, Busan 49241, Republic of Korea; 2 Biomedical Research Institute, Pusan National University Hospital, Busan 49241, Republic of Korea; 3 Department of Obstetrics and Gynecology, Pusan National University School of Medicine, Busan 49241, Republic of Korea

**Keywords:** ovarian cancer, preoperative tumor markers, CT, MRI

## Abstract

**Simple Summary:**

Preoperative tumor markers and imaging often differ in predicting whether an ovarian tumor is malignant. We evaluated the correlation between the predictive values of imaging and tumor markers for diagnosing ovarian tumors, especially when there were discrepancies between the two. We found that, to prevent misidentification, it is necessary to understand the underlying disease that can increase tumor marker levels or benign tumors, which are difficult to distinguish from malignant tumors in imaging tests and several rare histopathologies with low risk of ovarian malignancy algorithm values. The malignant prediction of tumor markers and imaging was different in many patients with ovarian tumors, and this study will help to reduce the misidentification of tumors.

**Abstract:**

Preoperative tumor markers and imaging often differ in predicting whether an ovarian tumor is malignant. Therefore, we evaluated the correlation between the predictive values of imaging and tumor markers for diagnosing ovarian tumors, especially when there were discrepancies between the two. We enrolled 1047 patients with ovarian tumors. The predictive values and concordance rates between the preoperative risk of ovarian malignancy algorithm (ROMA) and imaging, including CT and MRI, were evaluated. Diagnoses of 561 CT (77.9%) and 322 MRI group (69.2%) participants were consistent with the ROMA. Among them, 96.4% of the CT (541/561) and 92.5% of the MRI (298/322) group predicted an accurate diagnosis. In contrast, 67.3% (101/150) of CT and 75.2% (100/133) of MRI cases accurately predicted the diagnosis in cases with discrepancies between ROMA and CT or MRI; a total of 32% (48/150) of the CT and 25.5% (34/133) of the MRI group showed an accurate ROMA diagnosis in cases with discrepancies between ROMA and imaging. In the event of a discrepancy between ROMA and imaging when ovarian tumor malignancy prediction, the question is which method should take precedence. This study demonstrates that MRI has the greatest diagnostic accuracy, followed by CT and ROMA. It is also important to understand underlying diseases and benign conditions and rare histopathologies of malignant tumors.

## 1. Introduction

Ovarian cancer is the eighth most common cancer diagnosed and remains a major cause of cancer-related death among women. Distinguishing between malignant and benign ovarian tumors is important for preserving reproductive function in young patients and improving survival. The diagnosis of ovarian cancer typically relies on a combination of imaging and tumor markers.

Serum CA125 levels and transvaginal ultrasound have been used in the differential diagnosis of adnexal masses [1]; however, there is a broad consensus that CA-125 should not be used as an ovarian cancer disease screening tool. Its role in the initial evaluation of adnexal masses remains debatable; according to the recent national guidelines of adnexal mass management [2], CA-125 levels may be elevated in both malignant and benign conditions [2]. HE4 was found to be a reliable biological marker for detecting ovarian cancer [3]. CA-125 levels were more sensitive but less specific than HE4 markers alone, according to the French National College of Obstetricians and Gynecologists (CNGOF). CNGOF also concluded that complementary studies are necessary before using HE4 routinely [4]. The combination of HE4 and CA125 values resulted in an algorithm to assess the malignancy of the ovarian tumor, the risk of ovarian malignancy algorithm (ROMA), which could provide high sensitivity and specificity for the early detection of ovarian cancer [3].

Contrast-enhanced computed tomography (CT) is not a part of the management of strategy for adnexal masses [4,5,6,7]; however, it should be performed in women when malignant disease is suspected [8]. It is the current imaging modality for ovarian cancer staging and treatment follow-up [9,10]. However, CT does not have sufficient specificity for diagnosing ovarian cancer (overall sensitivity and specificity of CT were 0.79 and 0.87, respectively) [11]. Magnetic resonance imaging (MRI) is the modality of choice for characterizing indeterminate or large adnexal masses detected on ultrasound or CT with high sensitivity (83%), specificity (84%), and diagnostic accuracy (83%) [9,12].

In clinical practice, tumor markers and imaging, including CT and MRI, often differentially predict whether a tumor is malignant in each patient. In this study, we evaluated the concordance between imaging findings and tumor markers for the diagnosis of ovarian cancer. We also evaluated the cases of discrepancy between tumor markers and CT/MRI.

## 2. Materials and Methods

### 2.1. Study Design and Population

This was a retrospective, observational, and single-center study. Samples from patients at a gynecology clinic for symptomatic or suspected malignant ovarian cysts or pelvic tumors were collected between January 2017 and March 2021. Information on menopausal status, age, diagnosis, histologic type of tumor, and day of surgery was collected. Two pathologists histologically confirmed the status of the surgically resected ovarian tumor tissue using the International Federation of Gynecology and Obstetrics (FIGO) ovarian staging system.

A total of 1047 patients (693 premenopausal patients (median age: 35, range: 13–54 years) and 354 postmenopausal patients (median age: 61, range: 46–84 years)) were enrolled. Histopathological types of tumors among the enrolled patients are described in Table 1. Of the enrolled patients, 79.2%, 6.4%, and 14.3% had benign, borderline, and malignant tumors, respectively.

### 2.2. Testing of Tumor Markers

Laboratory tests were performed within 1–2 weeks prior to surgery. Samples were collected using a serum separator tube and centrifuged within 30 min after arrival at the laboratory. Elecsys^®^ assays for CA125 and HE4 (Roche Diagnostics, Basel, Switzerland) were performed using a Cobas^®^ e602 analyzer (Roche Diagnostics) for electrochemiluminescence immunoassay. The quantifiable ranges were 0.6–5000 U/mL for CA125 and 15–1500 pmol/L for HE4. The ROMA values for premenopausal and postmenopausal women were calculated using the results of the Elecsys^®^ CA125 and HE4 assays. The calculated predictive value (PI) was inserted into the equation to calculate the ROMA value. The PI was calculated separately for premenopausal and postmenopausal patients. The ROMA value (%) was calculated as exp(PI)/[1 + exp(PI) × 100], where exp(PI) = ePI. PI (premenopausal) was calculated as –12.0 + 2.38 × ln [HE4] + 0.0626 × ln [CA125], and PI (postmenopausal) was calculated as –8.09 + 1.04 × ln [HE4] + 0.732 × ln [CA125]. The recommended cutoff levels were CA125, 35 U/mL; HE4 (premenopausal), 92.1 pmol/L; HE4 (postmenopausal), 121 pmol/L; ROMA (premenopausal), 11.4%; and ROMA (postmenopausal), 29.9%, according to the manufacturer’s instructions. If the patient’s CA125 and HE4 levels were above the cutoff, they were described as high. If the patient’s ROMA value was above the cutoff, they were described as having a high risk of malignancy, and if the patient’s ROMA value was below the cutoff, they were described as low risk.

### 2.3. Imaging

Pelvic CT and/or MRI scanning were performed for benign, borderline, or malignant prediction. A total of 418 premenopausal and 164 postmenopausal patients underwent only CT; 214 premenopausal and 113 postmenopausal patients underwent only MRI; and 61 premenopausal and 77 postmenopausal patients underwent both CT and MRI. Patients who underwent CT scans were divided into a CT group and those who underwent MRIs into an MRI group. Patients who underwent both CT and MRI were included in both groups.

Because of the long timeframe of patient enrollment from 2017 to 2021, multiple imaging systems were utilized. We used to a 64-detector row CT scanner (Discovery 750 HD, Discovery CT 750 HD; GE Healthcare, Milwaukee, WI, USA), a 256-detector row CT scanner (Revolution; GE Healthcare) for CT scanning and 1.5-T system (Magnetom Avanto and Symphony, Siemens Medical Solutions, Erlangen, Germany), a 3-T MR system (Magnetom Trio, Siemens Medical Solutions), Signa Premier 3T (GE Healthcare), and Signa Architect 3T (GE Healthcare) in this study.

### 2.4. Statistical Analysis

When calculating the accuracy, positive predictive value (PPV), and negative predictive value (NPV), inconclusive cases were excluded. Numerical variables did not follow a normal distribution and were presented as medians and ranges, and groups were compared using the Mann–Whitney U test. *p*-values < 0.05 were considered statistically significant. All the analyses were performed using the MedCalc statistical software V.20.106 (MedCalc Software, Ostend, Belgium).

## 3. Results

### 3.1. Concordance Rate between ROMA and CT/MRI

A total of 561 cases (379 (79.1%) premenopausal and 182 (75.5%) postmenopausal) and 322 cases (191 (69.4%) premenopausal and 131 (68.9%) postmenopausal) were consistent with ROMA and imaging in the CT (720 patients) and MRI groups (465 patients), respectively (Table 2).

### 3.2. High ROMA Value and Benign Prediction in Imaging

Cases of 81 and 58 ovarian tumors with a high ROMA value and benign prediction in the CT and MRI groups, respectively, are described in Table 3. Overall, 14.3% (27/188) and 19.3% (17/88) of premenopausal and 12.5% (1/8) and 20% (1/5) of postmenopausal patients with endometriosis showed a high ROMA value in the CT and MRI groups, respectively. Furthermore, 8.4% (12/143) and 6.7% (3/45) of premenopausal and 11.5% (3/26) and 33.3% (2/6) of postmenopausal patients with mature cystic teratomas showed a high ROMA value in the CT and MRI groups, respectively.

Renal dysfunction was found as the underlying condition of chronic kidney disease in all patients with pathologically confirmed benign ovarian tumors, very high HE4 (>300 pmol/L) levels, low CA125 (35 U/mL) values, and a benign imaging prediction (5 patients of the CT group and 1 patient of the MRI group).

### 3.3. Low ROMA Value and Malignant Prediction in Imaging

Cases of 69 and 75 ovarian tumors with low ROMA value and malignant prediction in the CT and MRI groups, respectively, are described in Table 3. A total of 11 borderline tumors (including 8 mucinous type) and 18 malignant tumors (including 6 mucinous carcinomas, 3 clear cell carcinomas, and 3 adult granulosa cell tumors) showed low ROMA values in the CT group. In the MRI group, 24 borderline tumors (including 15 mucinous type) and 24 malignant tumors (including 6 mucinous carcinomas, 4 clear cell carcinomas, and 5 adult granulosa cell tumors) showed low ROMA values.

### 3.4. Predictive Values of Tumor Markers and Imaging

ROMA distinguished benign from malignant tumors with an overall prediction accuracy of 79.6% (834/1047). CT and MRI distinguished benign from malignant tumors, with an overall prediction accuracy of 90.3% (642/711) and 87.2% (398/456), respectively. The PPV values for ROMA, CT, and MRI were 51.5%, 66.4%, and 81.8%, respectively, and the NPV values for ROMA, CT, and MRI were 88.9%, 96.1%, and 91.2%, respectively.

**Figure 1:**
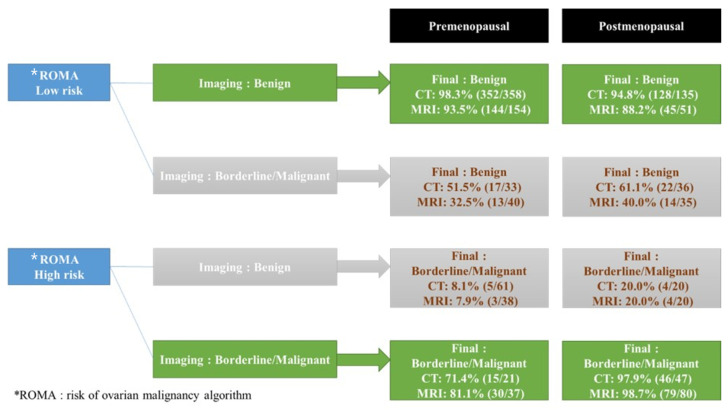
Predictive values of final diagnosis in ROMA, CT, and MRI in cases consistent with the ROMA and imaging and cases with discrepancies between ROMA and CT or MRI. Nine patients in the CT group and ten patients in the MRI group were excluded from this analysis due to inconclusive CT or MRI interpretations shown in Table 2. * ROMA: risk of ovarian malignancy algorithm.

Consistent with the ROMA and imaging, 96.4% (541/561) of the CT group and 92.5% (298/322) of the MRI group predicted an accurate diagnosis. In contrast, 67.3% (101/150) of the CT group and 75.2% (100/133) of the MRI group predicted accurate diagnosis in cases with discrepancies between ROMA and CT or MRI, respectively; a total of 32% (48/150) of the CT group and 25.5% (34/133) of the MRI group showed an accurate ROMA diagnosis in cases with discrepancies between ROMA, CT, or MRI, and 11.1% and 12.0% concordance cases with a high ROMA value and benign prediction on imaging were confirmed as malignant tumors in the CT and MRI groups, respectively. In contrast, 56.0% and 36% of cases with low ROMA values and malignant predictions on imaging were confirmed as benign tumors in the CT and MRI groups, respectively (Figure 1).

### 3.5. Distribution of ROMA

In cases with low ROMA values, there was no statistical difference in the ROMA values between patients with benign and malignant imaging predictions.

**Figure 2:**
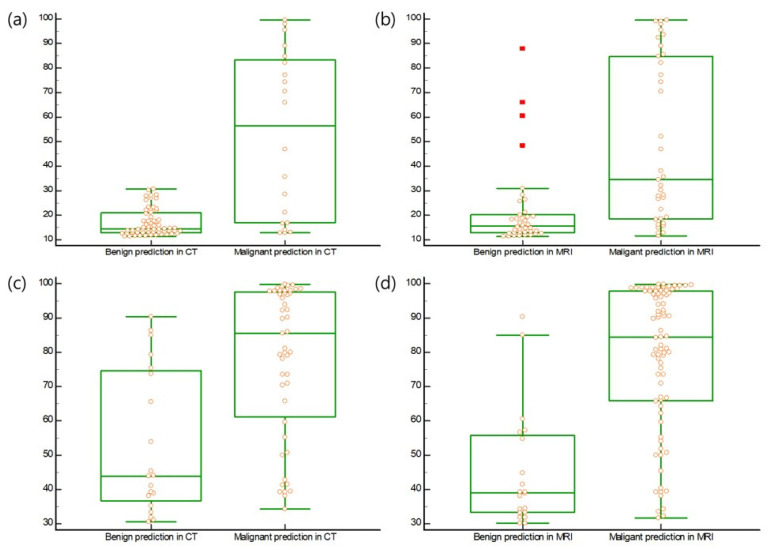
(**a**) ROMA values (median, 14.6; range, 11.4–30.8) in premenopausal patients with benign prediction of imaging were higher than those (median, 56.4; range, 12.9–99.5) in premenopausal patients with malignant predictions in the CT group (*p* < 0.0001). (**b**) ROMA values (median, 15.6; range, 11.4–87.9) in premenopausal patients with benign prediction of imaging were higher than those (median, 34.6; range, 11.6–99.5) in premenopausal patients with malignant predictions in the MRI group (*p* < 0.0001). (**c**) ROMA values (median, 43.9; range, 30.6–90.3) in postmenopausal patients with a benign prediction of imaging were higher than those (median, 85.5; range, 34.2–99.7) in postmenopausal patients with malignant predictions in the CT group (*p* = 0.0001). (**d**) ROMA values (median, 39.0; range, 30.1–90.3) in postmenopausal patients with a benign prediction of imaging were higher than those (median, 84.5; range, 31.7–99.7) in postmenopausal patients with malignant predictions in the MRI group (*p* < 0.0001). All data was displayed on the boxplot by yellow circle dots. The values above the upper quartile plus three times the interquartile range were represented by a red square dot on the boxplot.

In cases with high ROMA values, ROMA values (median 14.6 and 15.6) in premenopausal patients with benign imaging predictions were higher than those (median; 56.4 and 34.6) in patients with malignant predictions in the CT and MRI groups, respectively (Figure 2) (*p* < 0.0001). ROMA values (median; 43.9 and 39.0) in postmenopausal patients with benign imaging predictions were higher than those (median; 85.5 and 84.5) in patients with malignant predictions in the CT and MRI groups, respectively (Figure 2) (*p* = 0.0001, *p* < 0.0001).

### 3.6. Discrepancies in Imaging Studies

In total, 138 patients (61 premenopausal and 77 postmenopausal patients) underwent both CT and MRI, and the interpretations of CT and MRI were concordant in 107 patients. Thirty-one patients with discrepancies between CT and MRI findings are presented in Table 4.

## 4. Discussion

Ovarian cancer consists of various histological types and is often detected at an advanced stage; therefore, it is important to accurately predict malignancy during the preoperative examination. However, in actual clinical practice, tumor markers and imaging, including CT and MRI, often differentially predict whether a tumor is malignant. In this study, only 69.2–77.9% of the cases showed consistent results with the ROMA and imaging in patients with ovarian tumors, and 92.5–96.4% of patients with consistent results with the ROMA and imaging had accurately predicted diagnoses; however, 67.3% (101/150) of CT cases, 75.2% (100/133) of MRI cases, and 25.5–32% of cases with the ROMA accurately predicted diagnoses in case of discrepancies between the ROMA and imaging.

CA-125 levels may be elevated in several types of ovarian cancer, including epithelial cell tumors, carcinosarcomas, teratomas, and secondary ovarian malignancies [13]. Among epithelial ovarian cancers, serous carcinomas most frequently manifest elevated CA-125 levels [14]. Grandi et al. reported that the sensitivity of CA-125 was significantly higher in the diagnosis of stage I endometrioid ovarian cancer versus all other subtypes (72.4% vs. 49%) [15]. However, the diagnosis of clear cell tumors such as granulosa cell tumors and dysgerminomas is also complicated, and CA-125 levels are typically not effective at distinguishing between benign and malignant tumors [13]. HE4 is increasingly being used to identify certain subtypes of epithelial ovarian cancers, particularly serous and endometrioid tumors. HE4 levels are elevated in up to 93% of serous ovarian tumors [16,17]. In most patients with endometrioid ovarian tumors, the CA-125 and HE4 levels are elevated. The combination of elevated CA-125 levels, elevated HE4 levels, and a history of ovarian endometriosis or endometrial carcinoma should raise clinical suspicion of endometrioid ovarian adenocarcinoma [13]. In women younger than 40 with an adnexal mass, the Royal College of Obstetricians and Gynecologists recommends obtaining additional tumor marker measurements, including human chorionic gonadotropin, lactate dehydrogenase, and alpha–fetoprotein, due to rare forms of ovarian cancer, such as germ cell tumors of the ovary, and sex cord-stromal tumors that commonly present in younger age groups [6,7]. In this study, over 80% of the patients with serous and endometrioid ovarian tumors showed high ROMA values, whereas approximately 40–60% of patients with mucinous, clear cell, and germ cell ovarian tumors showed low ROMA values. Over 70% of the patients with granulosa cell tumors showed low ROMA values.

Karacan et al. reported eight cases in which patients underwent surgery due to increased serum HE4 levels and high ROMA values and in whom the final pathology was reported as benign, despite ultrasonography and MRI findings showing features of “typical” endometrioma [18]. Young women with endometrioma should undergo surgery based on elevated HE4 levels and high ROMA values, despite being at low risk for malignancy according to imaging findings. The main limitation of CA-125 as a diagnostic marker is its low specificity, particularly in premenopausal women. In this study, 12.5–20% of the patients with a high ROMA value and benign prediction of imaging had endometriosis. Cho et al. and Ustunyurt et al. reported that 8.4% and 23.3% of patients with mature cystic teratomas showed elevated CA125 levels, respectively [19,20], because mature cystic teratomas contain various tissues originating in the parthenogenesis of the oocyte. In this study, 8.4% of the patients with mature cystic teratomas showed high ROMA values.

A previous study showed that serum HE4 levels were significantly elevated in patients with chronic kidney disease [21] because HE4 could be expressed in the distal convoluted tubules of the kidney [22], and renal dysfunction may lead to the diminution of HE4 clearance [21]. This study identified chronic kidney disease in all patients with pathologically confirmed benign ovarian tumors with high HE4 (>300 pmol/L) and low CA125 and benign imaging predictions.

Numerous subtypes of ovarian cancers differ in terms of their cell line, mode of origin, growth speed, and feasibility of early detection [23]. Hence, imaging findings can help predict malignancy through features suggestive of malignancy (e.g., lesion size, wall/septal thickness, papillary projections, lobulated mass, necrosis, and solid and cystic architecture) [24]. Benign ovarian tumors are more likely to show mild enhancement and fewer ascites than borderline and malignant ovarian tumors in MRI findings [25]. Benign ovarian tumors usually exhibit thin-walled cysts and no solid components. However, borderline ovarian tumors display irregular thickened walls and less solid portions, and malignant ovarian tumors are more frequently characterized as solid or predominantly solid masses on MRI [25]. An MRI is frequently helpful in the further characterization of adnexal masses as the signal intensity reflects the pathologic characteristics of the lesion. However, it is possible that there could be a difference in readings between radiologists.

The limitation of this study was that it was a retrospective, single-center study. In addition, the use of different CT and MRI scanners may have influenced the results of this study. Different scanners may influence the interpretation of imaging findings in the cardiovascular system; however, differences in pelvic imaging have not been reported, making it impossible to discuss the implications.

## 5. Conclusions

In clinical practice, ROMA and imaging studies are commonly used to differentiate ovarian cancer from other pelvic masses preoperatively. Clinicians need to be careful when evaluating pelvic masses prior to surgery due to a significant discrepancy in a large percentage of ovarian cancer prediction methods. According to the present study, only 69.2–77.9% of patients with ovarian tumors had results that were consistent with ROMA and imaging, which was based on a single hospital’s population. Clinical situations that many cause discrepancies should also be ruled out. Moreover, endometriosis and chronic kidney disease must be considered when only elevated ROMA values and benign imaging are present. In cases with low ROMA values and only malignant predictive imaging, it is prudent to suspect the presence of a tumor with a mucinous or rare histopathology, thereby requiring an additional tumor marker.

In this study, 92.5–96.4% of patients’ diagnoses have been accurately predicted when ROMA and imaging results were consistent. Nonetheless, in cases of discrepancy between the ROMA and imaging, 67.3% of CT cases, 75.2% of MRI cases, and 25.5–32% of ROMA cases correctly predicted the diagnosis. In the case of a discrepancy between serum markers and imaging studies, the clinical challenge is determining which method should be prioritized. Figure 1 demonstrates that the prediction rate was highest for MRI, then CT, and then ROMA. In order to reach clearer conclusions, a large-scale prospective research is required.

## Figures and Tables

**Table 1:** Histopathologic types of tumors in the enrolled patients.

		Histopathologic Types	Number
Benign	Non-neoplastic mass	Endometrioma	272
(*N* = 830)		Simple or follicular or hemorrhagic cyst	26
		Corpus luteal cyst with/without endometriosis	7
		Paratubal cyst with/without endometriosis	28
		Other ^(a)^	14
	Epithelial ovarian tumor	Mucinous	123
		Serous	84
		Clear cell	1
		Mixed	18
	Germ cell tumor	Mature cystic teratoma	216
	Sex cord-stromal tumor	Thecoma–fibroma	39
		Leydig cell tumor	2
Borderline	Epithelial ovarian tumor	Mucinous	43
(*N* = 67)		Serous	17
		Endometrioid	2
		Clear cell	1
		Mixed	4
Malignant (*N* = 150)	Epithelial ovarian tumor	Mucinous	16
		Serous	76
		Endometrioid	10
		Clear cell	15
		Mixed	4
	Germ cell tumor	Dysgerminoma	1
		Immature teratoma	4
		Mixed	1
	Sex cord-stromal tumor	Granulosa cell tumor	11
	Metastatic cancer		
	Other cancer		12

^(a)^ Other includes actinomycosis (*N* = 1), endosalpingiosis (*N* = 2), hydrosalpinx (*N* = 4), inflammation (*N* = 1), salpingitis (*N* = 2), struma ovarii (*N* = 2), tubo-ovarian abscess (*N* = 2).

**Table 2:** Predicting in imaging and ROMA in premenopausal and postmenopausal patients with ovarian tumors.

			CT Group		MRI Group	
			Pre ^(a)^	Post ^(b)^	Pre ^(a)^	Post ^(b)^
Predicting in Imaging	ROMA	Final Diagnosis	(*N* = 479)	(*N* = 241)	(*N* = 275)	(*N* = 190)
Benign	Low	Benign	352	128	144	45
		Borderline	4	5	6	5
		Malignancy	2	2	4	1
	High	Benign	56	16	35	17
		Borderline	4	0	3	0
		Malignancy	1	4	0	3
Borderline or Malignancy	Low	Benign	17	22	13	14
		Borderline	4	7	15	9
		Malignancy	12	7	12	12
	High	Benign	6	1	7	1
		Borderline	1	4	3	5
		Malignancy	14	42	27	74
Inconclusive	Low	Benign	3	2	1	1
		Borderline	1	0	1	2
		Malignancy	2	0	0	0
	High	Benign	0	1	1	0
		Borderline	0	0	2	1
		Malignancy	0	0	1	0

^(a)^ Pre: Premenopausal state, ^(b)^ Post: Postmenopausal state.

**Table 3:** Final diagnosis of cases with prediction discrepancy between ROMA and imaging.

			High ROMA Value/ Benign Predicting in Imaging				Low ROMA Value/ Malignant Predicting in Imaging			
			CT Group		MRI Group		CT Group		MRI Group	
	Final Diagnosis	Histological Types	Pre ^(a)^	Post ^(b)^	Pre ^(a)^	Post ^(b)^	Pre ^(a)^	Post ^(b)^	Pre ^(a)^	Post ^(b)^
Benign	Non-neoplastic mass	Endometrioma	27	1	17	1	5	2	1	
		Simple or follicular or hemorrhagic cyst	1	2	1			1		1
		Corpus luteal cyst with/without endometriosis	1		3					
		Paratubal cyst with/without endometriosis	1	1	1					
		Other	3 ^(c)^		3 ^(d)^	2 ^(e)^				
	Epithelial ovarian tumor	Mucinous	6	1	6	4	6	10	7	6
		Serous	3	4	1	1	3	3	1	
		Clear cell				1				
		Mixed	1	1		1		3		1
	Germ cell tumor	Mature cystic teratoma	12	3	3	2	3		3	
	Sex cord-stromal tumor	Thecoma–fibroma	1	3		5		3		5
		Leydig cell tumor					1		1	1
Borderline	Epithelial ovarian tumor	Mucinous	3		1		3	5	10	5
		Serous			1		1	1	5	1
		Endometrioid								
		Clear cell								1
		Mixed	1		1			1		2
Malignant	Epithelial ovarian tumor	Mucinous					5	1	4	2
		Serous						1	1	1
		Endometrioid		2		1				1
		Clear cell		1		1		3		4
		Mixed	1	1						
	Germ cell tumor	Dysgerminoma					1		1	
		Immature teratoma					2		2	
		Mixed								
	Sex cord-stromal tumor	Granulosa cell tumor				1	2	1	3	2
	Metastatic cancer						1			
	Other cancer							1	1	2

^(a)^ Pre: Premenopausal state, ^(b)^ Post: Postmenopausal state ^(c)^ chronic salpingitis, acute and chronic inflammation, hydrosalpinx. ^(d)^ Actinomycosis, acute and chronic inflammation, acute salpingitis. ^(e)^ Struma ovarii, hemorrhagic infarction.

**Table 4:** Case review in the interpretation of prediction discrepancy between CT and MRI.

		MRI				
		Benign	Benign or Borderline	Borderline	Borderline or Malignancy	Malignancy
CT	Benign		2 (Benign 1 Borderline 1)	2 (Borderline 2)		4 (Benign 1 Malignancy 3)
	Borderline	3 (Benign 3)				3 (Borderline 1 Malignancy 2)
	Borderline or malignancy	3 (Benign 2 Borderline 1)	1 (Borderline 1)	1 (Benign 1)		1 (Malignancy)
	Malignancy	12 (Benign 9 Borderline 1 Malignancy 2)	1 (Benign 1)	4 (Benign 2 Borderline 1 Malignancy 1)	2 (Benign 2)	
	Inconclusive	2 (Benign 2)				1 (Benign 1)

## Data Availability

The data presented in this study are available on request from the corresponding author.
